# Evaluation of the AnnAGNPS Model for Predicting Runoff and Nutrient Export in a Typical Small Watershed in the Hilly Region of Taihu Lake

**DOI:** 10.3390/ijerph120910955

**Published:** 2015-09-02

**Authors:** Chuan Luo, Zhaofu Li, Hengpeng Li, Xiaomin Chen

**Affiliations:** 1 College of Resources and Environmental Sciences, Nanjing Agricultural University, No. 1, Weigang Road, Xuanwu District, Nanjing 210095, Jiangsu, China; E-Mails: lc_notek@163.com (C.L.); xmchen@njau.edu.cn (X.C.); 2 Nanjing Institute of Geography and Limnology, Chinese Academy of Sciences, No. 73, Beijing East Road, Nanjing 210095, Jiangsu, China; E-Mail: hpli@niglas.ac.cn

**Keywords:** AnnAGNPS model, nitrogen, parameter sensitivity analysis, phosphorus, runoff

## Abstract

The application of hydrological and water quality models is an efficient approach to better understand the processes of environmental deterioration. This study evaluated the ability of the Annualized Agricultural Non-Point Source (AnnAGNPS) model to predict runoff, total nitrogen (TN) and total phosphorus (TP) loading in a typical small watershed of a hilly region near Taihu Lake, China. Runoff was calibrated and validated at both an annual and monthly scale, and parameter sensitivity analysis was performed for TN and TP before the two water quality components were calibrated. The results showed that the model satisfactorily simulated runoff at annual and monthly scales, both during calibration and validation processes. Additionally, results of parameter sensitivity analysis showed that the parameters Fertilizer rate, Fertilizer organic, Canopy cover and Fertilizer inorganic were more sensitive to TN output. In terms of TP, the parameters Residue mass ratio, Fertilizer rate, Fertilizer inorganic and Canopy cover were the most sensitive. Based on these sensitive parameters, calibration was performed. TN loading produced satisfactory results for both the calibration and validation processes, whereas the performance of TP loading was slightly poor. The simulation results showed that AnnAGNPS has the potential to be used as a valuable tool for the planning and management of watersheds.

## 1. Introduction

Non-point source pollution occurs when rainfall or irrigation water runs over land or through the ground, picks up pollutants and deposits them into rivers, lakes, or coastal waters or introduces them into ground water [1]. It is an important environmental and water quality management problem, and non-point sources presently account for the majority of water quality problems [2]. The increases in nutrient losses and riverine nutrient loads have resulted in nuisance algal blooms, the depletion of dissolved oxygen, and other water quality impairments [3]. Severe problems with water quality seem to make it unlikely that the water body will continue to support aquatic life and human consumption [4].

Watershed modeling can be a valuable tool for studying the relationships between conditions and the quality of water in a watershed [5]. The modeling of environmental deterioration to better understand and manage natural resources, such as river basins and watersheds, is a continuous process. Basin scale models that incorporate weather data and watershed characteristics such as topography assist in the delineation of the watershed and are tools for the development of management strategies in a watershed and river basins [6]. In the last four decades, several hydrological and water quality models have been developed to assist in understanding hydrologic systems and pollutant loadings [7], such as AnnAGNPS (Annualized Agricultural Non-Point Source Pollution Model) [8], ANSWERS (Areal Non-point Source Watershed Environment Response Simulation) [9], SWAT (Soil and Water Assessment Tool) [10], and HSPF (Hydrological Simulation Program FORTRAN) [11]. These models can be used to simulate the transport processes of runoff, sediment, nutrients and other chemical substances. Detailed reviews of these models can be found in the literature [12,13,14].

The AnnAGNPS model combines the latest advances in GIS (Geographic Information System) data manipulation with physical characterization of the catchments, offering modeling opportunities for ungauged areas or for areas with limited data that prohibit the use of models relying on calibration for the derivation of input variables [15]. The model has been successfully used in many areas of the world in recent years, including Spain [16,17,18], Nepal [19], Italy [20], Canada [21], USA [22,23,24] and China [5]. These studies evaluated the ability of the AnnAGNPS model to predict runoff and pollutant loadings under different climate or land-use conditions in various watersheds with areas ranging from 0.1 to 130 km^2^. However, many of those studies have focused on evaluating the model’s suitability and on testing its performance regarding hydrologic and sediment transport estimation [16,17,18,19,20,21,22,23,24], and few efforts have been made to analyze the sensitive parameters for nutrient concentrations and to evaluate the model’s ability to predict them [4,15,25,26]. Thus, the purpose of this study is to validate the capability of the AnnAGNPS model to predict runoff and to analyze the sensitive parameters in regard to nutrient concentrations and evaluate the capability of the model to simulate nutrient loadings in a small watershed for a long time period (nine years).

## 2. Materials and Methods

### 2.1. AnnAGNPS Model Description

In this study, AnnAGNPS version 5.1 was applied. AnnAGNPS is a batch-process, continuous simulation, watershed-scale model designed to aid in the evaluation of long term, hydrologic and water quality responses to agricultural management practices [27]. It was jointly developed by the United States Department of Agriculture (USDA), the Agricultural Research Service (ARS) and the Natural Resources Conservation Service (NRCS) [28]. It consists of a system of computer models developed to predict NPS pollutant loadings within an agricultural watershed [16]. With the support of a routing system, continuous simulation has been realized [29]. The model simulates runoff, sediments, nutrients and pesticides, leaving the land surface and shallow subsurface and moving through the channel system to the watershed outlet, with output available atthe event, monthly and annual scales [30]. AnnAGNPS model is also designed to assist in determining BMPs (Best Management Practices), TMDLs (Total Maximum Daily Loads), and in risk cost/benefit analysis.

In AnnAGNPS, the analyzed watershed can be divided into many homogenous (in terms of soil type, land use and land management) cells or subwatersheds (up to 40 km^2^) to quantitatively estimate precipitation runoff and sediment, as well as nutrient and pesticide loadings [16,31]. The cells are irregular basins with comparatively uniform physical and hydrological characteristics, which allows for the analysis of any point within the watershed. The physical or chemical constituents are routed from each cell and are either deposited within the reaches or transported out of the watershed [4]. Cells and reaches and their topographic properties can be estimated by TOPAGNPS (Topographic Parameterization program used for AGNPS) and AGFLOW (Agricultural watershed Flownet generation program), which are additional modeling components in AnnAGNPS [32].

Surface runoff is estimated based on the Soil Conservation Service [33] Curve Number (CN) method. CN represents the runoff producing potential of soils, and has a range of 0–100. The Revised Universal Soil Loss Equation (RUSLE) [34] is used to estimate the daily sheet and rill erosion of the area. Considering that the RUSLE does not simulate the transport of eroded particles, the Hydro-Geomorphic Universal Soil Loss Equation (HUSLE) is used to simulate sediment delivery to the stream [35]. For N and P, a basic mass conservation equilibrium is employed to estimate nutrients generation and loading for rainfall events [36]. The model has a fixed N and P cycle, which considers the amounts of N and P joining or leaving the watershed [15].

### 2.2. Study Area

**Figure 1:**
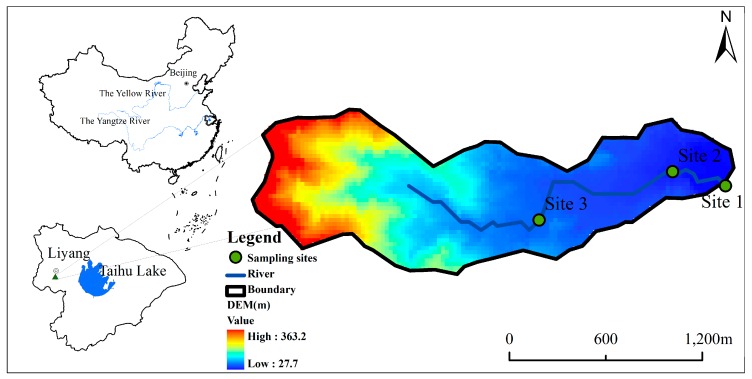
The Map of the Wucun watershed showing sampling sites, the digitized stream and the watershed boundary.

The study watershed is named Wucun and is located in the western basin of Taihu Lake in China. The area of the watershed is 1.81 km^2^ (181ha). The river originates from low hilly terrain with a maximum elevation of approximately 360 m, and it travels 2500 m while finding its way to the outlet (sampling stations), which is at an elevation of approximately 27 m (Site 1) (Figure 1). The watershed is characterized by a subtropical monsoon climate, with a mean annual rainfall of 1169.3 mm and an annual average temperature of 15.4 °C (2005–2013), and the rainy seasons mainly occur over 4–9 months. The soil type in the upstream of the watershed is generally Typic Ali-Udic Argosols (TAUA) and that in the downstream of the watershed is Typic Hapli-Stagnic Anthrosols (THSA). Properties of soils are presented in Table 1. The land use in this watershed is largely dominated by forest, and other land uses mainly include urban land and agriculture.

**Table 1:** Properties of soils in the study area.

Classification [37]	Hydrological	K Factor Value	Depth (mm)	Content (%)			
				Clay	Silt	Sand	Very Fine Sand
TAUA	B	0.04	0–16	15.12	41.98	42.9	3.01
			16–29	17.41	37.69	44.9	3.11
			29–56	16.84	40.26	42.9	3.01
THSA	C	0.04	0–14	19.22	53.06	27.72	1.97
			14–24	12.9	59.38	27.72	1.97
			24–36	12.73	57.3	29.97	2.12
			36–100	13.26	31.21	55.53	3.50

### 2.3. Data Acquisition

#### 2.3.1. Climate Data

Climate data required for running the AnnAGNPS model includes maximum temperature, minimum temperature, precipitation, dew point temperature, sky cover or solar radiation, and wind speed. In this study, the time span of climate data was nine years from January 2005 to December 2013. Data of maximum temperature, minimum temperature, and precipitation were downloaded from the Liyang national climate station (31°26′N, 119°29′E; at 7.7m height) for 2005 to 2010 and were provided by the Nanjing Institute of Geography and Limnology, Chinese Academy of Sciences from 2011 to 2013. Additionally, dew point temperature was calculated from the relative humidity and mean air temperature, and solar radiation was deduced from temperature and sunshine duration, as suggested by the China Meteorological Administration (CMA) [38].

#### 2.3.2. Topographic Data

This study utilized a 1:50,000 relief map, which was scanned and then digitized to construct a Digital Elevation Model (DEM) (30 m ×30 m). The DEM is used for topographic evaluation, drainage identification, watershed segmentation, and subcatchment parameterization.CSA (Critical Source Area) and MSCL (Minimum Source Channel Length) are employed to divide the watershed. The CSA value defines a minimum drainage area below which a permanent channel is defined, and the MSCL is the minimum acceptable length of the cell swale for the source channel to exist. Various combinations of CSA/MSCL values were tried for watershed delineation. Finally, values of 2 ha and 70 m were adopted to define CSA and MSCL, respectively. As a result, 78 cells and 36 reaches were obtained, and the cell area ranged from 428 to 157,940 m^2^.

#### 2.3.3. Soil Data

Running the AnnAGNPS model requires specific properties of all soil layers. The physical properties include particle size distribution, bulk density, saturated hydraulic conductivity, field capacity, wilting point, *etc.* Chemical properties such as pH, organic matter, organic and mineral nitrogen and phosphorous were considered. The digital soil map obtained from the Soil Survey Office in Jiangsu Province of China provided little information about the properties of soil. Thus, parts of properties such as saturated hydraulic conductivity, field capacity and wilting point were calculated by the Soil Water Characteristics (SWCT) module of the SPAW (Soil Plant Atmosphere Water) model [39]. Additionally, the soil erodibility factor (K) was derived following Wischmeier and Smith [40]. The soil properties for various types of soil in the study area are shown in Table 1.

#### 2.3.4. Land Use Data

**Figure 2:**
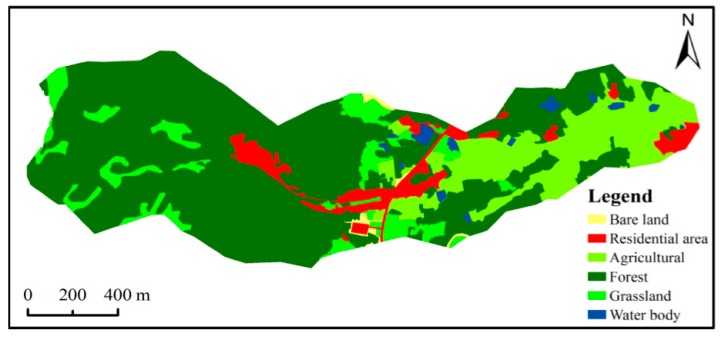
Land use map of the Wucun watershed.

The original land use map was obtained from an aerial image taken in 2009 with a resolution of 0.5 m × 0.5 m. The image data of the study watershed was reclassified into six types of land uses by visual interpretation. As shown in Figure 2, most of the study area was covered by forest and agricultural land use types with proportions of 66% and 17%, respectively. The remaining area was covered by the bare land, urban land, grass land and water body land-use types. The agriculture is rotated between rice and rape. The information concerning the management schedule (Table 2) was obtained by interviewing local farmers.

**Table 2:** Schedules of annual cultivation and agricultural practices in the Wucun watershed.

Land Use	Date	Operation	Observation
Rice	21 May	Seeding	Combined seeding machine
	12 June	Fertilization	Carbamide
	10 July	Fertilization	Ammonium bicarbonate
	10 August	Fertilization	Compound fertilizer
	25 August	Weeding	Triadimefon/Diniconazole
	3 September	Fertilization	Compound fertilizer
	18 October	Grain harvesting	5000–6000kg·ha^−1^
	25 October	Tillage	Moldboard
Rape	1 November	Seeding	Combined seeding machine
	10 December	Fertilization	Carbamide
	5 February	Fertilization	Carbamide
	10 March	Fertilization	Carbamide
	11 March	Fertilization	Ammonium bicarbonate
	10 May	Grain harvesting	2200–3000kg·ha^−1^
	18 May	Tillage	Moldboard

#### 2.3.5. Hydrologic and Nutrient Loading Data

Due to the lack of long-term runoff records in the Wucun watershed, the realistic annual runoff was estimated by an empirical formula of rainfall–runoff [41], which was developed in the similar hilly region of the Taihu Lake watershed. Thus, the simulated annual runoff at Site 1 (Figure 1) could be compared with the estimated one to calibrate and validate the AnnAGNPS model. Regarding the climate data, data from 2005–2009 were used for annual calibration, and data from 2010 to 2013 were used for annual validation.

Monthly water samples (total nitrogen and total phosphorus) at three monitoring sites (see Figure 1) were sampled and then analyzed in the laboratory during December 2012 to December 2013. Monthly runoff data were also measured at this period. Thus, the nutrient loadings were calculated by multiplying nutrient concentrations by monthly runoff. The monthly runoff could be calibrated at Site 1, while Site 2 and Site 3 were employed to validate the simulation processes. For nutrients, there was a similar process to that of monthly runoff. Site 1 was chosen to calibrate the nutrients, and Site 2 and Site 3 were used for validation. This calibration and validation method is similar to a random spot check, which may more accurately reflect the efficiency of the model.

### 2.4. Parameter Sensitivity Analysis

Sensitivity analysis is a methodological study of the response to the selected output variables to variations in parameters and driving variables [5]. It has been widely applied in hydrological models such as SWAT [42,43] and HSPF [44] to help users identify crucial parameters.

#### 2.4.1. Runoff Parameter

Most of the worldwide studies evaluating AnnAGNPS [19,22,30,45] showed that CN is the most sensitive parameter to surface runoff prediction, and these studies were successfully calibrated for surface runoff simulation by adjusting CN values. Therefore, in this study, we also make the simulated runoff approximate the actual runoff by adjusting the values of CN.

#### 2.4.2. Nutrients parameters

The nutrient parameters used in some literature [4,15,31,46] are not clear. These studies predicted nutrients by using the AnnAGNPS model, but the correlative parameters that they used were not reported. Additionally, some previous studies had made some contributions to analyzing the sensitivity parameters for nutrients [5,47,48]. Yuan *et al.* [47,48] indicated that the Initial nitrogen concentration in the soil, Plant uptake and Fertilizer mixing code were sensitive parameters for nitrogen loading, and Initial soil *p* contents as well as *p* application rate were sensitive parameters for phosphorus loading. Liu [5] concluded that CN, Rainfall quantity, Fertilizer application and Fertilizer available were sensitive parameters for both total nitrogen (TN) and total phosphorus (TP).

In this study, sensitivity analysis was considered necessary, and was carried out before calibration for nutrients. Parts of the parameter values were obtained from the survey data, such as Initial soil *N* and *p* contents, as mentioned above. We mainly wanted to evaluate the effects of human factors on nutrient simulation, including the fertilizer and crop parameters Residue Mass Ratio, Root Mass, Canopy Cover, Fertilizer Rate, Fertilizer Depth, Fertilizer Inorganic, and Fertilizer Organic. Based on a literature review [16,49,50], Differential Sensitivity Analysis (DSA) was employed to evaluate the sensitivity due to its simplicity and low-need for computation time. The DSA calculate one point in the parameter space by adjusting the parameter with a fixed percentage while the other factors remain constant. As shown below, each selected parameter would be changed by an increment of ∆x = ±10%, ±20%, ±30%, ±40%, ±50%, ±60% while fixing the values of the other parameters. The gradient of the output response with respect to the selected parameter was used to quantify the degree of sensitivity I. Each term of ∆x would have a value of I′, and I was defined by the average of the six terms. *I*′ was computed as follows:

(1) $$I\prime =\frac{\left(y_{2}-y_{1}\right)/y_{0}}{2△x/x_{0}}$$

The model output *y*_0_ is calculated with an initial value *x_0_* of the parameter *x*; *x*_0_ is varied by ±∆*x*, yielding *x*_1_= *x*_0_−Δ*x* and *x*_2_ = *x*_0_+ Δ*x*; and *y*_1_ and *y*_2_ correspond to *x*_1_ and *x*_2_.

The Sensitivity Index is ranked into four categories [51] as follows: less than 0.05—small to negligible sensitivity; 0.05–0.2—medium sensitivity; 0.2–1.0—high sensitivity; and more than 1.0—very high sensitivity.

### 2.5. Evaluation of Model Performance

The results of the simulation were analyzed for “goodness-of-fit” with the observed data. The performance evaluation of AnnAGNPS was carried out in a comprehensive manner, as suggested by Legates and McCabe *et al*. [20,52,53]. The coefficient of determination (*R*^2^), coefficient of efficiency (*E*), root mean square error (RMSE), and coefficient of residual mass (CRM) were employed for model assessment. Additionally, it should be noted that *R*^2^ and E are overly sensitive to extreme values, which may mislead the evaluation of model performance. To avoid this, a revised coefficient of efficiency was defined as *E*′, which could reduce the effect of squared terms [52].The formulas for these coefficients are listed in following.

The coefficient of determination ranges from 0 to 1, with higher values indicating better agreement and is given as:

(2) $$R^{2}={\left\{\frac{\sum_{i=1}^{N}\left(O_{i}-\bar{O}\right)\left(S_{i}-\bar{S}\right)}{\sqrt{\sum_{i=1}^{N}{\left(O_{i}-\bar{O}\right)}^{2}}\sqrt{\sum_{i=1}^{N}{\left(S_{i}-\bar{S}\right)}^{2}}}\right\}}^{2}$$

The coefficient of efficiency *E* is given as:

(3) $$E=1-\frac{\sum_{i=1}^{N}{\left(O_{i}-S_{i}\right)}^{2}}{\sum_{i=1}^{N}{\left(O_{i}-\bar{O}\right)}^{2}}$$

*E* is dimensionless and ranges from minus infinity to 1. As proposed by Van [54], the results are highly satisfactory for an *E* value equal or larger than 0.75, satisfactory between 0.36 and 0.75, and unsatisfactory for an *E* value smaller than 0.36.

The modified coefficient of efficiency is calculated as:

(4) $$E\prime =1-\frac{\sum_{i=1}^{N}\left|O_{i}-S_{i}\right|}{\sum_{i=1}^{N}\left|O_{i}-\bar{O}\right|}$$

In general, E′ has a lower value than *E*, and the model can be considered satisfactory when E′ ranges from 0.51 to 0.71 [20].

The root mean square error (RMSE) is given as:

(5) $$RMSE=\sqrt{{\frac{\sum_{i=1}^{N}\left(O_{i}-S_{i}\right)}{N}}^{2}}$$

The coefficient of residual mass (CRM) is given as:

(6) $$CRM=\frac{\sum_{i=1}^{N}O_{i}-\sum_{i=1}^{N}S_{i}}{\sum_{i=1}^{N}O_{i}}$$

where O*_i_* is the observed data, S*_i_* is the simulated data, $\bar{O}$ is the mean of the observed data set, $\bar{S}$ is the mean of the simulated data set, *i* is the *i*th event, and N is the number of observations.

## 3. Results and Discussion

During the nine-year study period, annual rainfalls ranged from 828.10 to 1267.50 mm with a mean and standard deviation of 1052.92 and 122.60 mm, respectively. At the monthly scale, only the runoff data from 2013 were available, and the recorded rainfalls were in the range of 10–136.60 mm with resulting runoff varying from 4.43 mm to 53.23 mm. Approximately 45% of rainfall was concentrated from May to July.

### 3.1. Runoff Calibration

**Figure 3:**
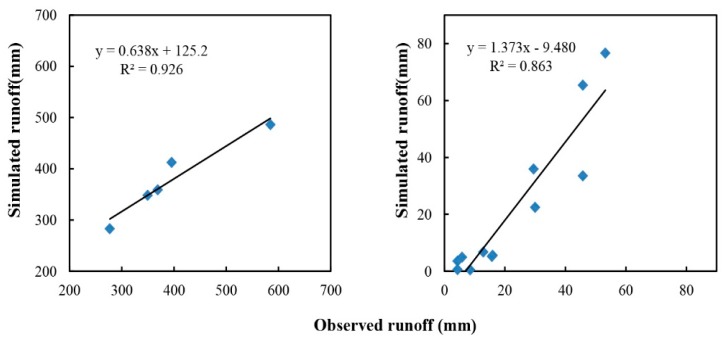
Comparison between observed and simulated runoff at annual (**left**) and monthly (**right**) scales for calibration process.

In watershed modeling, calibration and validation are important steps for ensuring the quality of model simulations. Upon completion of the data entry using the AnnAGNPS Data Input Editor, runoff was calibrated by adjusting CN for all landuse categories. The best results (Figure 3) were obtained by increasing the CN values of agriculture, forest, grassland and urban land by 20%, 30%, 20%, 20%, respectively (Table 3). Before calibration, *E* and *E*′ both had negative values, and the CRMs had high values (Table 4). These parameters indicated that substantial differences existed between simulations and observations. However, acceptable statistical parameters were obtained after calibration processes. As shown in Table 4, at annual scale, the difference between simulated and observed average annual runoff was very small (as indicated by small CRMs), and the other statistical parameters included *R*^2^ = 0.93, *E* = 0.81, and *E*′ = 0.65. At the monthly scale, the statistical analyses showed that *R*^2^ = 0.86, *E* = 0.59, *E*′ = 0.39 and CRM = 0.04.

Monthly scale simulation was barely satisfactory. Some months were overestimated, whereas some were underestimated. The main reason for this is that the monthly runoff data were calculated from the measured data when sampling in the middle of each month. This process may lead to a large difference for the actual flow, especially in the rainy or dry periods, and it may yield a smaller or larger result when estimating the monthly runoff based on this value.

**Table 3:** Initial and final CN values for each land-use type.

Land Use	Curve Numbers for Hydrologic Soil Groups							
	Initial Values				Final Values			
	A	B	C	D	A	B	C	D
Bare land	65	75	82	95	65	75	82	95
Residential area	55	68	79	85	66	82	95	99
Agricultural	60	72	80	85	78	86	96	99
Forest	48	60	73	80	58	78	95	96
Grass land	42	69	79	87	50	83	95	98

**Table 4:** Estimated statistical parameters of model performance for default and calibration/validation.

Items	Calibration					Validation				
	*R*^2^	*E*	*E*′	RMSE	CRM	*R*^2^	*E*	*E*′	RMSE	CRM
Annual scale										
Run off	0.93(0.76) *	0.81(−5.32)	0.65(−2.23)	45.09(257.83)	0.05(0.63)	0.88(0.82)	0.86(−8.00)	0.65(−2.2)	26.95(213.62)	−0.02(0.58)
Monthly scale										
Run off	0.86(0.55)	0.59(−3.21)	0.39(−1.33)	11.24(21.58)	0.04(0.62)	0.84(0.75)	0.65(−2.64)	0.42(−1.75)	10.65(20.31)	0.02(0.59)
						0.87(0.78)	0.81(−1.63)	0.62(−0.55)	8.71(15.16)	0(0.45)
TN	0.91(0.77)	0.86(0.33)	0.71(0.12)	100(180.25)	0.22(0.87)	0.92(0.85)	0.71(0.29)	0.58(0.08)	130.85(210.34)	0.42(0.92)
TP	0.66(0.38)	0.37(−1.05)	0.46(0.10)	1.67(3.15)	0.14(0.92)	0.62(0.28)	0.18(−1.26)	0.37(−0.55)	1.18(2.67)	0.19(0.88)

* Numbers in parenthesis are default values.

### 3.2. Runoff Validation

**Figure 4:**
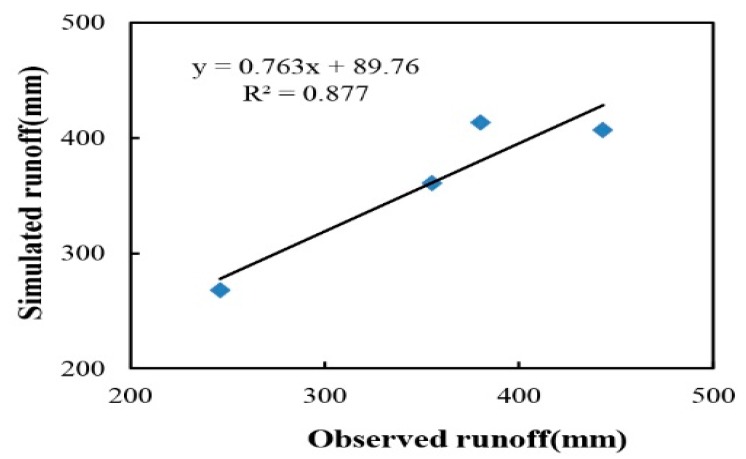
Comparison between observed and simulated runoff at the annual scale for the validation process.

**Figure 5:**
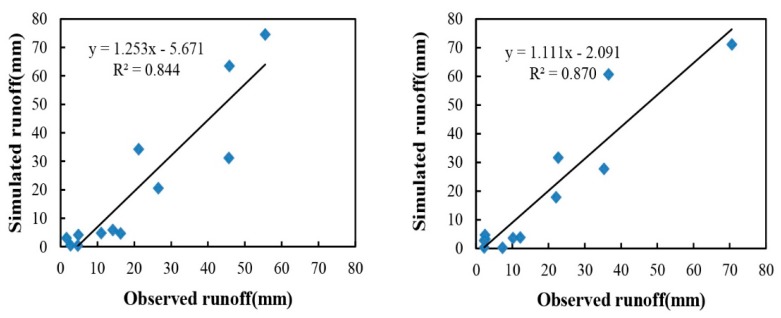
Comparison between observed and simulated monthly runoff at Site 2 (**left**) and Site 3 (**right**) during validation process.

Validation was carried out after calibration. At the annual scale (Figure 4), the difference between simulated and observed runoff was only approximately 2.6%, with averages of 361.98 mm and 356.34 mm, respectively, and with *E* = 0.86, *E*′ = 0.65, and CRM = −0.02. At the monthly scale (Figure 5), two sites were employed to validate the simulation process, with values of 0.65 and 0.81 for *E*, and 0.42 and 0.62 for *E*′ at Site 2 and Site 3, respectively. Both of these results confirm the ability of the model to predict runoff after calibration.

Site 3 performed more efficient than Site 2 during the validation process. In the geography, Site 3 is upstream of Site 2, and thus the watershed based on Site 3 as the outlet is smaller than that based on Site 2. As noted by Chahor and Taguas [16,17], the smaller the watershed is, the more satisfactory the model prediction seems to be. Additionally, the cultivated areas mainly concentrate between Site 2 and Site 3, and agricultural irrigation is also a factor affecting the validation accuracy of Site 2. Nevertheless, Site 2 is still in the range of required accuracy for the model evaluation. In the validation process, both of the two sites yield satisfactory results, thus confirming the accuracy of the calibration process.

### 3.3. Nutrient

#### 3.3.1. Results of the Parameter Sensitivity Analysis

**Figure 6:**
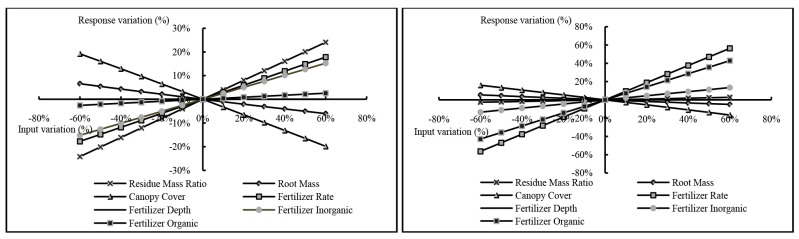
The sensitivity of nutrient for (**left**) TN and (**right**) TP to the selected input parameters

Figure 6 depicts the relationship between input variation and output variation, where most of the selected parameters had a linear effect on nutrient loading prediction. Fertilizer rate, Fertilizer organic, Fertilizer inorganic, and Residue mass ratio had positive correlations (Pearson Correlation = 1, *p* < 0.01) with the nutrient loading model output, while the Root mass and Canopy cover had negative correlations (Pearson Correlation = −1, *p* < 0.01). This might be explained by more fertilizer leading to more nutrient loss; otherwise, more vegetation could reduce nutrient loss.

According to Lenhart [51], Fertilizer rate, Fertilizer organic, Canopy cover and Fertilizer inorganic can be classified as high sensitive parameters for TN output, whereas Residue mass ratio, Fertilizer rate, Fertilizer inorganic, and Canopy cover are highly sensitive parameters for TP output. The remaining parameters have small to negligible sensitivity and medium sensitivity (Table 5).

**Table 5:** Sensitivity classification of AnnAGNPS input parameters to nutrient loading.

Input Parameters	Sensitivity Index for TN	Sensitivity Index for TP
Residue mass ratio	0.04	0.39
Root mass	−0.09	−0.10
Canopy cover	−0.26	−0.32
Fertilizer rate	0.94	0.30
Fertilizer depth	0.00	0.00
Fertilizer inorganic	0.25	0.26
Fertilizer organic	0.69	0.04

#### 3.3.2. Nutrient Calibration

**Figure 7:**
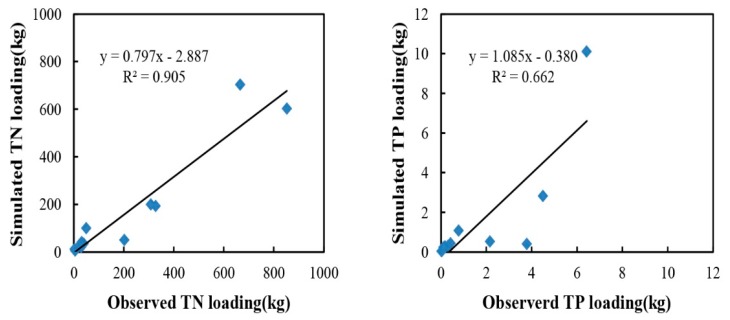
Comparison between observed and simulated TN (**left**) and TP (**right**) loading during the calibration process.

Similar to runoff calibration, nutrient calibration was also made for Site 1. Due to the lack of nutrient data, only monthly TN and TP loadings were investigated in this study. Based on the result of parameters sensitivity analysis, calibration was first carried out by adjusting the most sensitive parameters, and then adjusting the medium-sensitivity parameters. Several efforts were made before obtaining the final result. Figure 7 (left) shows the plot of simulated *versus* observed TN loadings with regression. The value of CRM was 0.22 > 0, indicating under-prediction and yielding an *R*^2^ value of 0.91. The coefficient of efficiency and modified coefficient of efficiency for TN loadings showed satisfactory results of 0.86 and 0.71 (Table 4), respectively. Moreover, TP loading was also slightly under-predicted (CRM = 0.14). As shown in Figure 7 (right), the coefficient of determination (*R*^2^) was found to be 0.66, which meant that the model was only able to explain or represent approximately 66% of the varieties in the observed data. The results for *E* and *E*′ were 0.38 and 0.46, respectively.

#### 3.3.3. Nutrient Validation

**Figure 8:**
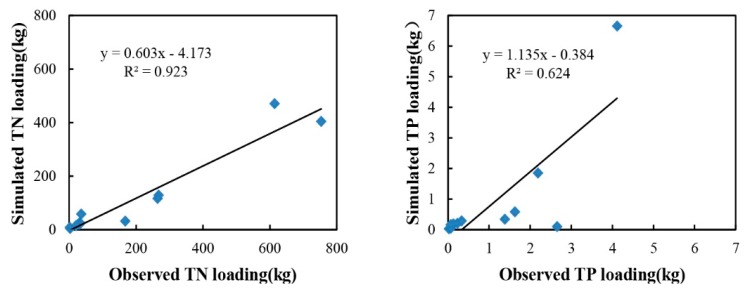
Comparison between observed and simulated TN (**left**) and TP (**right**) loading during the validation process.

The validation process was performed for Site 2 and Site 3 (Figure 1). Figure 8 shows the plot of simulated *versus* observed loading for TN and TP in Site 2. TN prediction performed well, with an R^2^ value of 0.92 (Figure 8, left) and a CRM value of 0.42. The coefficient of efficiency and modified coefficient of efficiency for TN loading also showed satisfactory results of 0.71 and 0.58, respectively. These statistical values suggested that the model was able to correctly predict the site conditions. For the TP, *R*^2^ was 0.624 (Figure 8, right), the results for *E* and *E*′ were slightly poor at 0.18 and 0.37, respectively, but the CRM of 0.19 was acceptable (Table 4). The largest difference occurred in October, where the observed and simulated TP loading were 2.66 kg and 0.09 kg, respectively.Clearly, they made a substantial contribution to the low performance of TP. Although the TP prediction was not satisfactory, it was still able to represent a certain portion of the observed data.

The model performed satisfactorily for TN simulation at Site 2. Otherwise, TP simulation was poor at this site. Under-predicted values for TP clearly existed in the calibration and validation processes (Figure 7 and Figure 8). A lack of reliable information may have leaded to this underestimation, as information such as plant uptake and other natural phosphorus cycling were taken from unofficial sources.Many vital input parameters are needed for calibration purposes. The performance at Site 3 was poor, as large differences were observed in both TN and TP validation processes. The mean values of simulated and observed TN loading were 0.56 kg and 143.5 kg, and those of TP were 0.007 kg and 0.96 kg, respectively. The reason may be that Site 3 was located in the urban area, its concentrations of TN and TP were deeply affected by the residential activities, and the samplings from this site would yield high values. Thus, this site was not suitable for the calibration or validation processes.

Compared with previous studies applying the AnnAGNPS model to simulate nutrients, this study obtained similar or better results. Shamshad [15] reported the application of the AnnAGNPS model to a watershed (63.09 km^2^) with conditions and climate typical of Malaysia. In their evaluation of the model performance, the model performed satisfactorily for runoff simulation. However, poor statistical parameters were obtained for TN and TP simulation processes. The results of Pease’s simulation [4] also showed low performance for nutrient simulation in an east-central North Dakota watershed with an area of 1697 km^2^. Baginska [25] evaluated the model in a 2.55 km^2^ watershed of Currency Creek, Australia, and concluded that the model produced satisfactory results for event flows, but it yielded a high degree of uncertainty for nitrogen. Thus, it can be seen that the model had experienced low nutrient simulation performance at various scales. Additionally, the low performance of AnnAGNPS in predicting nutrient loading is also reported in other studies [31,55,56]. Due to the mechanism of the AnnAGNPS model where nutrient loading is based on mass conservation, any missing input or output information of nutrients in watershed will considerably affect the results. Furthermore, Bingner [36] noted that the model assumed that there was no tracking of nutrients from one day to the next, which means that there will definitely be a loss of mass. Additionally, a lack of reliable information and data regarding nutrients is a common phenomenon in many areas, and this may further contribute to the low performance of nutrient prediction. More detailed data should be monitored to obtain a more realistic watershed simulation.

## 4. Conclusions

The AnnAGNPS model was applied in a small agricultural watershed called “Wucun” in the upstream of the Taihu watershed to validate its capability to predict surface runoff and to test its capability to predict nutrient loading using data recorded from January 2005 to December 2013.

The model was calibrated in the period of 2005–2009 to achieve the best-fit runoff prediction, as runoff has a major impact on nutrient prediction. This was completed by adjusting the CN values. Then, the model was validated in the period of 2010–2013. The result was evaluated for “goodness-of-fit” between predicted and observed data using five statistical measures, namely the root mean square error (RMSE), coefficient of residual mass (CRM), coefficient of determination (*R*^2^), coefficient of efficiency (*E*) and modified coefficient of efficiency (*E*′). The values of the five parameters indicated a good correlation between the predicted and observed data, which suggested that the model possessed an adequate capability to simulate surface runoff.

Concerning nutrient loading, a parameter sensitivity analysis was first carried out to evaluate the sensitivity of the nutrient parameters. Fertilizer rate, Fertilizer organic, Canopy cover and Fertilizer inorganic were more sensitive to TN output, and Residue mass ratio, Fertilizer rate, Fertilizer inorganic and Canopy cover were more sensitive to TP output. The AnnAGNPS model was then calibrated based on the sensitivity analysis results. TN simulation produced satisfactory results for both the calibration and validation processes, whereas TP loading performance was slightly poor. Though the results were not very good, the model was still able to represent a certain portion of variability in the observed data. Generally, the study found that the AnnAGNPS model was qualified as a watershed modeling tool.
